# Shielding Properties of Epoxy Matrix Composites Reinforced with MgO Micro- and Nanoparticles

**DOI:** 10.3390/ma15186201

**Published:** 2022-09-06

**Authors:** M. I. Sayyed, Sabina Yasmin, Nouf Almousa, Mohamed Elsafi

**Affiliations:** 1 Department of Physics, Faculty of Science, Isra University, Amman 11622, Jordan; 2 Department of Physics, Chittagong University of Engineering and Technology, Chattogram 4349, Bangladesh; 3 Department of Physics, College of Science, Princess Nourah Bint Abdulrahman University, P.O. Box 84428, Riyadh 11671, Saudi Arabia; 4 Physics Department, Faculty of Science, Alexandria University, Alexandria 21511, Egypt

**Keywords:** gamma-rays, nanoparticles, epoxy, MgO, attenuation coefficient, particle size effect

## Abstract

The aim of the current study is to investigate the impact of introducing micro- and nanoparticle MgO as a filler into epoxy resin on the radiation shielding abilities of the prepared samples. To this end, we performed a gamma-radiation spectroscopy experiment with the help of an HPGe detector and Am-241, Cs-137, and Co-60 sources. We evaluated the particle size effect (PSE) and detected the maximum PSE value with the addition of 50 wt% MgO particles, indicating that nanoparticle MgO was more successful in shielding against incoming radiation than microparticle MgO. We compared the half-value layer (HVL) for the samples with 10 wt%, 20 wt%, and 30 wt % micro-MgO and nano-MgO and found that the HVL values were lower for the nanoparticle samples than for the microparticles samples, confirming that smaller particle sizes enhanced the shielding ability of the samples against radiation. The MFP results showed that epoxy matrices containing micro-MgO, for all investigated energies, resulted in higher MFP values that those containing nano-MgO.

## 1. Introduction

With the continued use of radiation technology, it is important to contain radiation and prevent it from coming into contact with humans and the environment to the greatest extent possible. Long-term exposure to ionizing radiation can lead to serious health hazards, which is why radiation shields are often used to prevent any harmful effects [1,2,3,4]. Radiation shields are specially designed materials that are placed between a source of radiation and an area that needs to be protected to absorb as much radiation as possible according to the specifications of the application. Depending on the circumstances, a radiation shield may need to be light, thin, non-toxic, or able to contain radiation for extended periods of time [5,6,7,8].

Epoxy resins are a good candidate for radiation shields owing to their low cost, high strength, low toxicity, resistance to corrosion, excellent mechanical properties, and low shrinkage. [9,10,11] These characteristics make epoxy composites a desirable shield to attenuate neutrons, X-rays, and gamma rays. For example, epoxy is especially effective against neutrons due to its hydrogen-dense structure. The properties of epoxy can be further enhanced by introducing filler particles, both micro- and nanoparticles, into an epoxy matrix [12,13].

Many approaches have been taken to further understand the properties of modified epoxy composites against ionizing radiation. Chang, L. et al. [14] studied the effect of introducing tungsten into epoxy composites and tested the gamma-ray shielding ability of the samples with increasing concentrations of tungsten introduced into the matrix. The results demonstrated that increasing tungsten content in the composites improved the shielding ability of the samples at the cost of the bending strength of the material. Aldhuhaibat, M. et al. [9] tested Al_2_O_3_ epoxy and Fe_2_O_3_ epoxy samples against high-energy gamma rays. These additives were chosen because of their relatively high atomic numbers, resulting in an improvement in the attenuation abilities of the composites with increasing filler content. Li, R. et al. [15] blended erbium oxide particles with basalt fibers to fabricate a basalt-fiber-reinforced plastic. The resulting mixture was used to reinforce the epoxy composite, and the results showed that the lightweight and mechanical properties of the three-phase composite made it useful for radiation shielding applications. Hashemi, S. et al. [10] also used a multistage manufacturing procedure to prepare a graphene oxide coating with Pb_3_O_4_, which was then added to epoxy resin and tested. Sahin, N. et al. [16] used a Yahyali Stone, a naturally abundant stone found in Turkey, to reinforce epoxy resin. The considerable amounts of Fe_2_O_3_, SiO_2_, and Al_2_O_3_ in the stone substantially enhanced the shielding abilities of the resulting composites. Additionally, many studies have centered around the effects of introducing tungsten oxide, by itself and with other fillers, into epoxy resin and investigating the resulting effects on the properties of the composite. In 2015, Chang et al. examined the effects of the amalgamation of tungsten elements on an epoxy matrix to identify the radiation shielding and mechanical features of epoxy resin. The radiation shielding properties of tungsten/epoxy composites increase with the increment of incorporated tungsten filler. An increase in the quantity of tungsten elements in the epoxy matrix amplifies the radiation protection ability of the tungsten/epoxy matrix [17]. The effect of nano- and micro-sized WO_3_ on epoxy composites was studied by Azman et al. in 2013, within the energy range of 10 KeV–100 KeV. The inclusion of nano-sized WO_3_ in epoxy composites resulted in an increased reduction capability compared to the inclusion of micro-sized WO_3_ in the energy range of 10 KeV–25 KeV. The size of WO_3_ in the epoxy matrix was associated with only negligible differences with respect to the shielding ability at higher energies in the range of 20 KeV–40 KeV. The inclusion of WO_3_ in the epoxy matrix enhanced the mechanical properties of the composite up to a certain concentration, with a negative effect on the mechanical properties associated with further addition of WO_3_ beyond that specific level [18]. In 2021, Cao et al. researched the gamma protection capacity of pure epoxy, Al_2_O_3_/epoxy, and Fe_2_O_3_/epoxy at energies of 0.662, 1.173, and 1.333 MeV. The gamma reduction ability of epoxy resins has been reported to increase with increased concentrations of the studied filler [19]. Karabul, Y. and Icelli, O. [20] prepared epoxy-based micro- and nano-Bi_2_O_3_- and -WO_3_-reinforced composites in an attempt to combine previous research on Bi_2_O_3_ epoxy composites with WO_3_ epoxy composites. After experimentally testing the composites, they found that the shields with high amounts of Bi_2_O_3_ and WO_3_ were highly effective against low-energy photons, which are typically found in medicinal applications. The nano-WO_3_ composites also had an advantage over their microparticle counterparts; however, this advantage was mostly present at lower energies. Azman, N. et al. [21] also investigated tungsten oxide epoxy composites; however, they focused on shielding against X-rays for uses in radiology and other fields of medicine. Hassan, M. et al. [22] introduced WB and WB_2_ microparticles treated with a coupling agent into an epoxy matrix. The WB_2_ epoxy samples were found to be better at absorbing gamma radiation than the WB samples.

In recent years, nanoparticle fillers have been increasingly tested, as opposed to previously used microparticle fillers, due to their high concentration of particles per gram. A smaller particle size leads to a more uniform distribution in the resin and an increased surface area to interact with, which often correlates with an improved shielding ability. This enhancement is especially noticeable at lower energies, where nanoparticle fillers tend to have a considerable advantage over their microparticle counterparts [23,24]. The aim of the current study is to investigate the effect of introducing micro- and nanoparticle MgO as a filler into epoxy resin on the radiation shielding abilities of the prepared samples.

## 2. Materials and Methods

### 2.1. Preparation 

The traditional casting method was used to form new samples to study the attenuation coefficients and their efficiency against photons. These samples consisted of a mixture of epoxy and magnesium oxide (MgO). A transparent epoxy resin and its hardener were selected, and micro and nano-sized MgO particles were used as filler. The raw materials were purchased from local stores. The purity of the micro-MgO particles was 97.8, with an average particle size of 60 ± 4 μm. MgO nanoparticles were purchased from Nano Tech company with a purity of 99.8 %, and according to TEM imaging, the average particle size was 20 ± 5 nm. The characteristics of micro- and nano-MgO were studied in [25]. The epoxy resin was a generic liquid type. For preparation, the mixture was added to an electric mixer after weighing each component and stirred well until it became homogeneous. Then, the homogeneous mixture poured into a plastic mold in the form of a disc and left for a whole day to dry and become solid.

**Figure 1:**
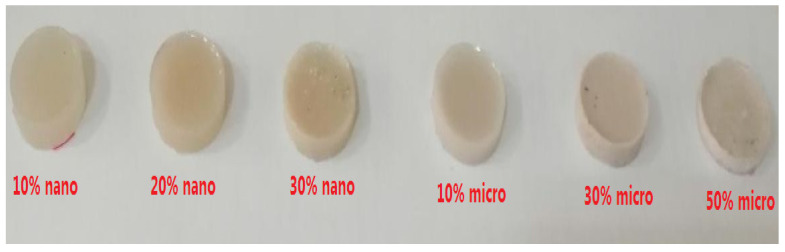
Epoxy-MgO composites were prepared in this study.

Samples were prepared according to the percentages mentioned in Table 1, with 2 g of hardener added for every 50 g of epoxy. Figure 1 shows the samples with 10 wt% to 50 wt% MgO microparticles and samples with 10 wt%, 20 wt%, and 30 wt% MgO nanoparticles (a homogeneous mixture was not achieved with the addition of 40 wt% and 50 wt% MgO nanoparticles).

### 2.2. Measurements

**Figure 2:**
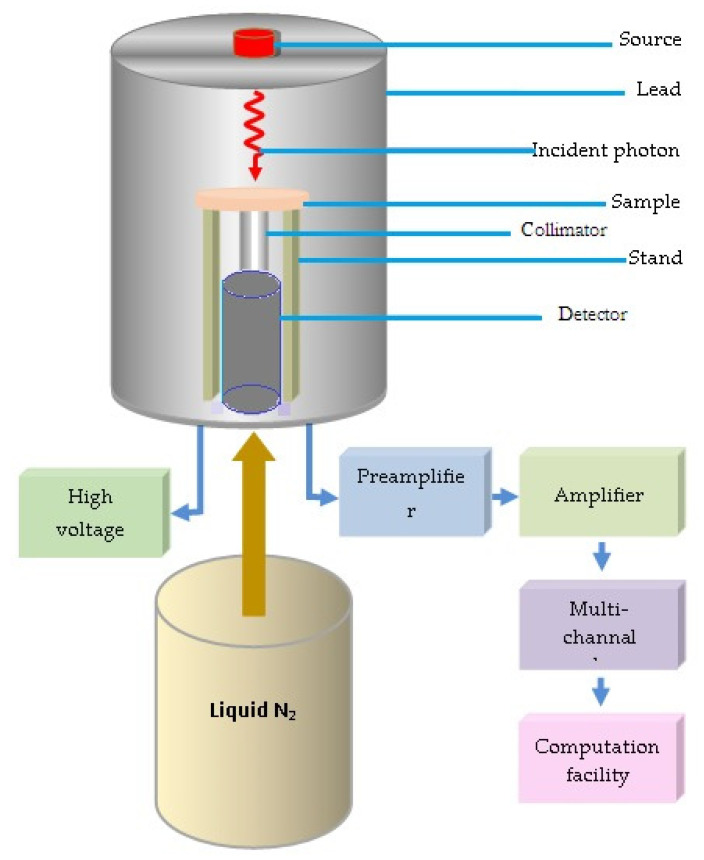
Schematic representation of the experimental work.

To experimentally measure the linear attenuation factor, HPGe detector and three radioactive point sources were used to obtain four energies covering low, medium, and high regions [26,27]. The sample was placed between the detector and the source in an appropriate position, as shown in Figure 2, and the count rate was calculated using a program connected to the device (Genie 2000 software) [28]. A sample of the thickness (*t*) was placed, and the count rate (*N*) was calculated; then, the sample was removed under the same conditions and arrangements, and the free count rate (*N*_0_) was calculated. The *LAC* was calculated experimentally using the following formula [29,30].

(1) $$LAC=\frac{1}{t}ln\frac{N_{0}}{N}$$

The experimental results of epoxy samples reinforced with MgO microparticles were compared with the XCOM results [24]. The mass attenuation coefficient (MAC) calculation was based on the compositions and concentrations of elements or oxides. The other shielding parameters for the samples reinforced with MgO micro- and nanoparticles were deduced from the *LAC* results and calculated according to protocols described in previous works [31,32,33,34,35,36].

## 3. Results and Discussion 

**Figure 3:**
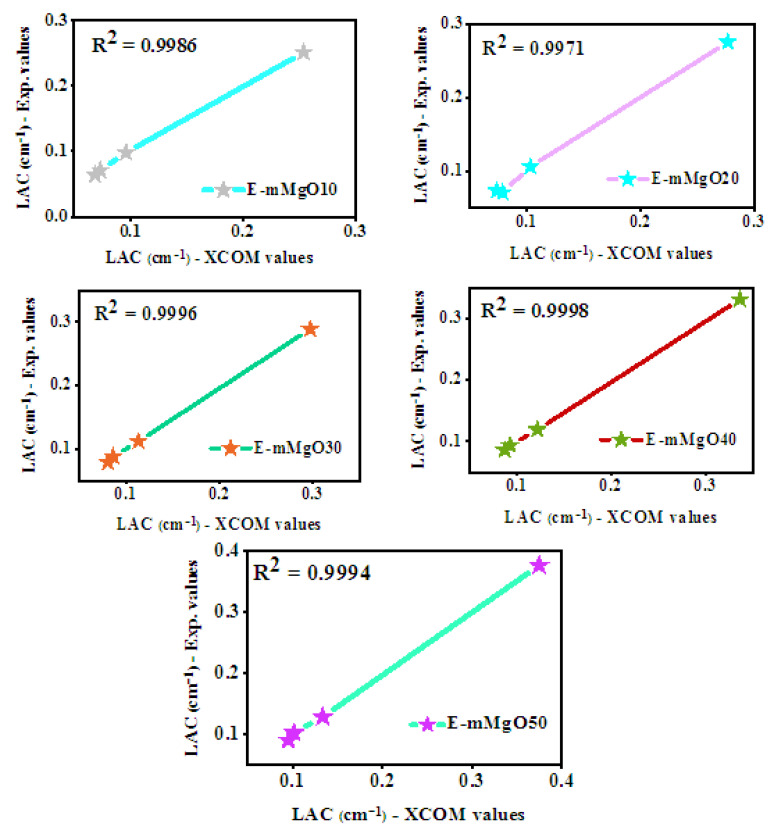
Graphical representation of XCOM versus experimental results of LAC values.

E-mMgO10, E-mMgO20, E-mMgO30, E-mMgO40, and E-mMgO50 epoxy matrix samples were prepared with 10, 20, 30, 40, and 50 wt% micro-MgO as filler material, respectively, to evaluate the effect on shielding properties against radiation. In this study, the experimental LAC values and the theoretical XCOM values for the micro-MgO epoxy samples were evaluated and compared. Figure 3 shows the experimentally obtained linear attenuation coefficient (*LAC*) values, as well as those simulated by XCOM software for E-mMgO10, E-mMgO20, E-mMgO30, E-mMgO40, and E-mMgO50 epoxy samples. The aim of this step was to validate the experimental setup. The value of R^2^ for all studied epoxy matrix samples was between 0.9971–0.9998, which is very close to 1, validating the precision of the experimental setup utilized in this study.

In order to elucidate the impact of the particle size of the filler material on the epoxy matrix with respect to radiation attenuation, the PSE factor was determined according to Equation (2), utilizing the same amount of nano- and microparticles:

PSE factor = µ_Nano__/_μ_Micro_ (2)

**Figure 4:**
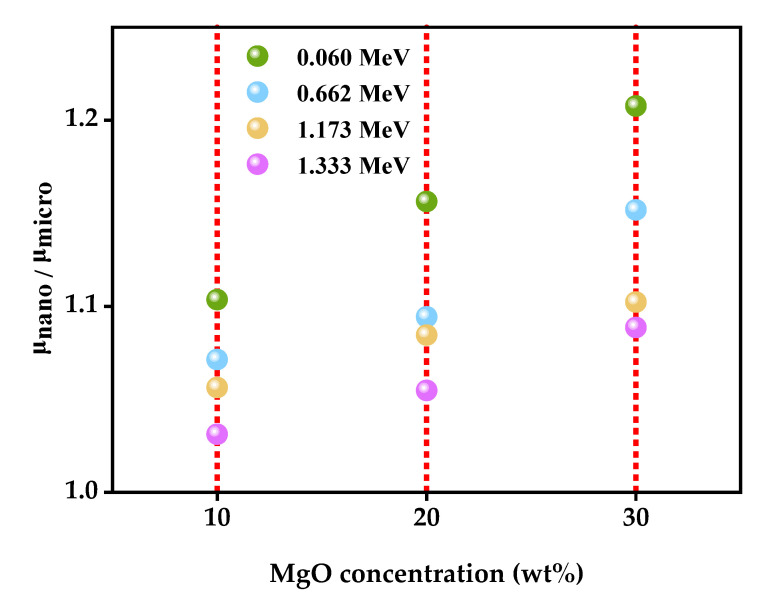
Change in PSE value the addition of micro-MgO and nano-MgO in the prepared epoxy matrix.

E-mMgO10, E-nMgO10, E-mMgO20, E-nMgO20, E-mMgO30, and E-nMgO30 epoxy matrix samples were prepared by adding 10 wt%, 20 wt%, and 30 wt% of micro-MgO and nano-MgO, respectively, to the epoxy matrix. At any incident photon energy level, a PSE value of more than one indicates that the inclusion of nanoparticles provides better shielding efficiency than the inclusion of the same amount of microparticles. Figure 4 shows that the PSE value exceeded one for every investigated concentration of MgO against 0.060, 0.662, 1.173, and 1.332 MeV photon energies, indicating that reducing the particle size of the filler (micro to nano size) but not changing the amount of filler resulted in increased absorption of incident photons [37]. Furthermore, the maximum value of the PSE factor was detected with the addition of the maximum tested concentration of MgO particles (50 wt%). Additionally, the PSE value increased with increased incident photon.

**Figure 5:**
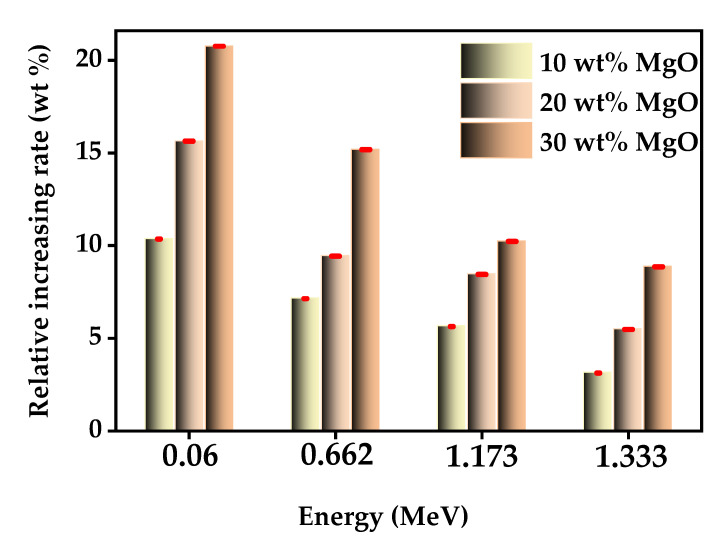
Relative increase (%) in the MAC of epoxy matrix samples with nano- and micro-MgO as filler.

Figure 5 shows the MAC of the epoxy matrix samples with nano- and micro-MgO as filler particles at energy levels of 0.060 MeV to 1.332 MeV. The relative rate of increase of MAC in samples containing nano-MgO was superior to that of samples with micro-MgO as filler. At 0.060 MeV, the value of MAC was higher in the epoxy matrix samples with 10 wt%, 20 wt%, and 30 wt% nano-MgO than those with 10 wt%, 16%, and 21% micro-MgO content, respectively. This result indicates that reducing the particle size of filler material from micro to nano enhanced the shielding ability of the prepared epoxy matrix samples. Furthermore, the rate of increase of the MAC value in the epoxy matrix samples with both nano- and micro-MgO decreased with increased photon energy, demonstrating particle size has a considerable impact on shielding ability in the low-energy range. 

**Figure 6:**
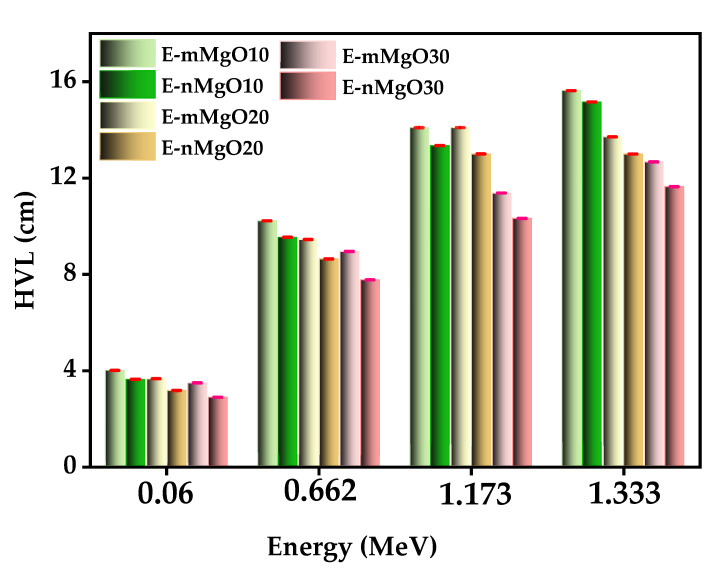
Comparison of HVL of epoxy matrix samples containing micro- and nano-MgO.

E-mMgO10, E-nMgO10, E-mMgO20, E-nMgO20, E-mMgO30, and E-nMgO30 epoxy matrix samples containing 10 wt%, 20 wt%, and 30 % micro-MgO and nano-MgO as filler were prepared to judge their shielding properties against radiation. We evaluated the impact of micro-MgO and nano-MgO on the epoxy matrix HVL; results are presented in Figure 6. The HVL value of sample E-mMgO10 was greater than that of sample E-nMgO10 at concentrations of 20 wt%, and 30 wt% micro-MgO and nano-MgO. Therefore, an epoxy matrix with nano-MgO provides superior radiation protection relative to an epoxy matrix containing micro-MgO, as evidenced by the lower HVL values of the nanoparticle samples relative to those of the microparticle samples. These findings indicate that reducing the particle size of the filler material enhanced the shielding ability of the samples against radiation. 

**Figure 7:**
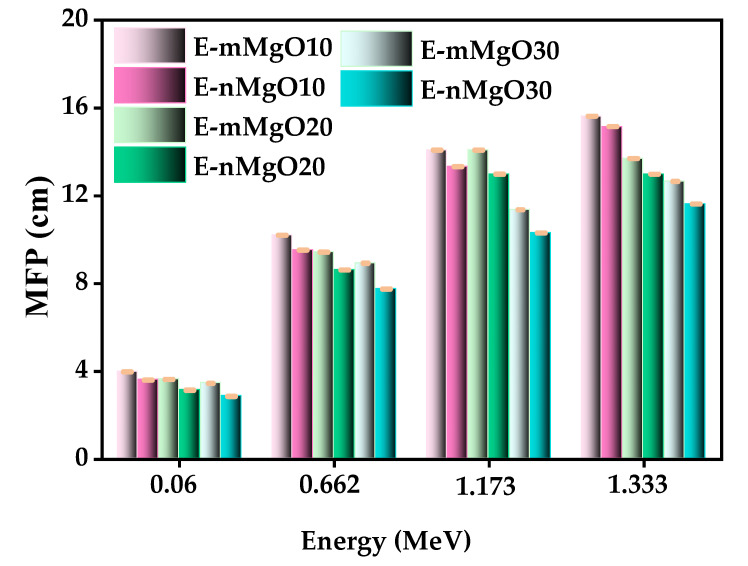
Mean free path of epoxy matrix samples with 10, 20, and 30 wt% micro-MgO and nano-MgO.

Figure 7 shows the MFP of three prepared epoxy matrices with 10, 20, and 30 wt% micro-MgO and nano-MgO at energies of 0.06 MeV, 0.356 MeV, 0.662 MeV, and 1.173 MeV. At all tested energy levels, the epoxy matrix samples containing micro-MgO had higher MFP values than those containing nano-MgO. In addition, the epoxy matrix sample with the highest MgO content had the lowest MFP of all tested samples. Moreover, all studied epoxy matrix samples containing nano-MgO had lower MFP values than those containing micro-MgO.

## 4. Conclusions

In this study, we tested six samples consisting of a mixture of epoxy and magnesium oxide (MgO) to assess the radiation-shielding ability of the samples against incident photons. We also evaluated the effect of size variation of MgO particles included in the epoxy resin. We combined concentrations of 10, 20, 30, 40, and 50 wt% micro-MgO with epoxy resin, as well as concentrations of 10 wt%, 20 wt%, and 30 wt% nano-MgO with epoxy resin. We compared experimentally obtained linear attenuation coefficient (*LAC*) values with those simulated by XCOM software to validate the experimental setup. Without changing the quantity of filler MgO filler particles, we observed PSE values > 1 at photon energies of 0.060 MeV, 0.662 MeV, 1.173 MeV, and 1.332 MeV, indicating that epoxy resins with nano-MgO filler had better defensive capabilities against radiation than epoxy resins with micro-MgO as filler. The nano-MgO epoxy samples had lower HVL values than the micro-MgO epoxy samples, indicating that a smaller particle size of the filler enhances the radiation defensive quality of the resulting epoxy composite. Moreover, epoxy matrices containing lower concentrations of MgO filler had lower MFP values, with lower MFP values associated with epoxy matrix samples containing nano-MgO filler as opposed to those containing micro-MgO. Therefore, an increase in the concentration of MgO filler enhances the defensive capacity of the epoxy matrix against incident radiation, and nano-sized additives enhance the shielding ability the matrix more than micro-sizes additives.

## Figures and Tables

**Table 1:** Percentages, codes, and densities of the prepared samples.

Percentages (wt%)			Density (g/cm^3^)		Micro Sample Codes	Nano Sample Codes
Epoxy	Micro-MgO Particles	Nano-MgO Particles	Micro	Nano		
90	10	10	1.182	1.188	E-mMgO10	E-nMgO10
80	20	20	1.277	1.279	E-mMgO20	E-nMgO20
70	30	30	1.389	1.395	E-mMgO30	E-nMgO30
60	40	—	1.522	—	E-mMgO40	—
50	50	—	1.683	—	E-mMgO50	—

## Data Availability

Not applicable.
